# Simple Bioparticle Filtration Device Based on an Ultralow-Fouling Zwitterionic Polyurethane Membrane for Rapid Large-Volume Separation of Plasma and Viruses from Whole Blood

**DOI:** 10.3390/membranes13050524

**Published:** 2023-05-17

**Authors:** Kun Wang, Hyang Seol, Alex Cheng, Nash McKeague, Megan Carlson, Wade Degraff, Sijia Huang, Sangil Kim

**Affiliations:** 1 Department of Chemical Engineering, University of Illinois at Chicago, Chicago, IL 60607, USA; 2 New Trier High School, New Trier, IL 60093, USA; 3 University of Chicago Laboratory Schools, Chicago, IL 60637, USA; 4 Lawrence Livermore National Laboratory, Livermore, CA 94550, USA

**Keywords:** plasma separation, portable plasma separation device, point-of-care device, viral load tests, zwitterionic membrane

## Abstract

Plasma separation from whole blood is oftent required as an essential first step when performing blood tests with a viral assay. However, developing a point-of-care plasma extraction device with a large output and high virus recovery remains a significant obstacle to the success of on-site viral load tests. Here, we report a portable, easy-to-use, cost-efficient, membrane-filtration-based plasma separation device that enables rapid large-volume plasma extraction from whole blood, designed for point-of-care virus assays. The plasma separation is realized by a low-fouling zwitterionic polyurethane-modified cellulose acetate (PCBU-CA) membrane. The zwitterionic coating on the cellulose acetate membrane can decrease surface protein adsorption by 60% and increase plasma permeation by 46% compared with a pristine membrane. The PCBU-CA membrane, with its ultralow-fouling properties, enables rapid plasma separation. The device can yield a total of 1.33 mL plasma from 10 mL whole blood in 10 min. The extracted plasma is cell-free and exhibits a low hemoglobin level. In addition, our device demonstrated a 57.8% T7 phage recovery in the separated plasma. The results of real-time polymerase chain reaction analysis confirmed that the nucleic acid amplification curve of the plasma extracted by our device is comparable to that obtained by centrifugation. With its high plasma yield and good phage recovery, our plasma separation device provides an excellent replacement for traditional plasma separation protocols for point-of-care virus assays and a broad spectrum of clinical tests.

## 1. Introduction

The global community continues to confront serious threats from a variety of virus diseases, including human immunodeficiency virus (HIV) [1], Ebola [2], hepatitis [3], and coronavirus disease 2019 (COVID-19) [4]. Blood viral loading testing plays a crucial role in mitigating and controlling bloodborne virus pandemics (e.g., HIV, malaria, syphilis, and Lyme disease) and monitoring the efficacy of virus disease treatments. For instance, more than 20 million people living with HIV are currently receiving antiretroviral therapy (ART), which requires continuous monitoring of HIV viral load in blood [1]. Although various viral load assay platforms exist for accurate, high-throughput viral load testing, these platforms usually require sophisticated laboratory instruments and highly trained laboratory technicians [1]. Meanwhile, diagnostic point-of-care (POC) tests have rapidly emerged and are expanding into laboratories and clinics [1,5], as these relatively new technologies possess great potential to decentralize and expand viral load testing, enhance efficiency in healthcare services, and improve viral suppression. However, plasma separation from whole raw blood is still required for POC viral load testing because unwanted cellular contents in the sample, such as hemoglobin and lactoferrin in blood cells, can interfere with nuclear acid polymerases and cause inaccurate quantification [6]. Currently, blood separation is typically performed in a diagnostic laboratory equipped with a high-speed centrifuge, which is not accessible for many less-developed regions [7,8,9].

Various POC plasma separation devices have been developed for on-site viral load testing. Devices can be classified into different categories based on the separation mechanism, including microfluidic separation [10,11,12,13], centrifugation [14,15,16], magnetic bead capture [17,18], and membrane-based separation [19,20,21,22]. Among these, microfluidic-based devices yield either highly diluted blood or only a small amount of plasma, ranging from a few to tens of microliters [23], and the complex structure of microfluidic-based devices hinders large-scale fabrication. In comparison, portable centrifugation devices based on egg-beater and fidget-spinner designs have stood out for their simplicity [14,15]. However, the plasma yield is still in the range of tens of microliters. To handle a higher number of blood samples, Vemulapati et al. developed a blood–plasma separator based on magnetic bead capture, which can yield 0.44 mL of plasma from 1 mL of whole blood in 45 s [17]. Nevertheless, the cost of this device and the storage of magnetic beads may hinder its application. Membrane-based separation provides a good alternative that is both simple and inexpensive. Liu et al. developed a membrane-based, sedimentation-assisted device that can extract 275 μL of plasma from 1.8 mL of whole blood in 7 min [19]. More recently, we also reported a plasma separation device consisting of an ultralow-fouling zwitterionic polymer-based membrane that yields 0.5–0.7 mL of plasma from 10 mL of whole blood [20]. However, the plasma yield is still unsatisfactory. For example, a state-of-the-art POC HIV assay with the desired sensitivity (<200 copies/mL) requires at least 1 mL of plasma for a single test [1,24]. A low sample volume may not possess sufficient target components for tests, thus giving inaccurate diagnosis results [25]. To the best of our knowledge, there have been no reports of a plasma separation device with a plasma yield larger than 1 mL for a single operation. Thus, a rapid, simple POC plasma separation device with a high-volume plasma output and high virus recovery has not yet been achieved.

This study aimed to fabricate a portable, simple-to-use, low-cost, disposable plasma separation device based on the high performance of an antifouling membrane that enables rapid large-volume plasma extraction from whole blood for POC virus assays. Our device benefits from a zwitterionic polyurethane-modified cellulose acetate (PCBU-CA) membrane, which can greatly inhibit the surface fouling of blood cells and membrane flux decline. Rapid plasma separation can be obtained with a simple three-step operation: blood injection, separation, and plasma collection. The utility of this separator for diagnostics was tested by separating plasma from whole blood spiked with a virus. The virus recovery in the plasma was characterized by a plaque assay and real-time polymerase chain reaction (RT-PCR). The device demonstrated a plasma yield of 1.3 mL from undiluted whole blood in 10 min with a ≈60% virus recovery. With its high-volume plasma yield and good virus recovery, this plasma separator has great potential for HIV load tests and a broad spectrum of POC virus assays.

## 2. Experimental Section

### 2.1. Materials

Phosphate-buffered saline (PBS) was purchased from Sigma-Aldrich (St. Lious, MO, USA). A cellulose acetate (CA) membrane with a cut-off size of 0.8 µm (average pore size of 0.4 µm) was obtained from Tisch Scientific (North Bend, OH, USA). Ethylenediaminetetraacetic acid (EDTA) anticoagulant-treated whole porcine blood was purchased from Sierra For Medical Science (Colton, CA, USA). T7 phage (BAA-1025-B2™) and *Escherichia coli* BL21 (BAA-1025) were purchased from the American Type Culture Collection (Manassas, VA, USA). A primer for T7 phage was ordered from Integrated DNA Technologies (Coralville, IA, USA). PowerUp™ SYBR™ Green Master Mix and DNase I solution were obtained from Thermo Fisher (Waltham, MA, USA).

### 2.2. Membrane Preparation and Characterization

**Figure 1:**
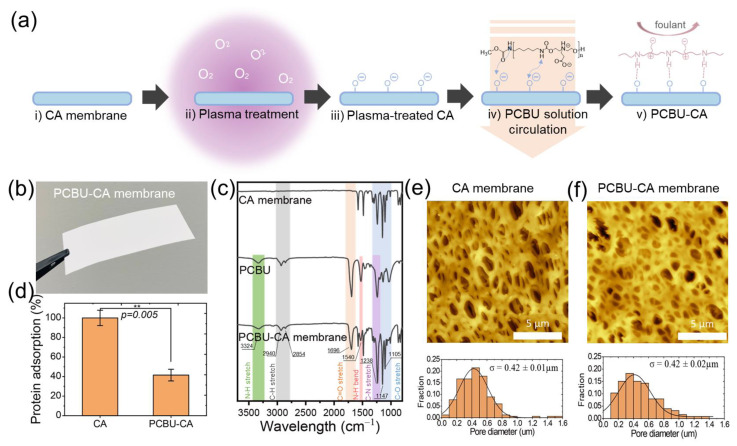
(**a**) Schematic representation of the PCBU coating process on a CA membrane for constructing an antifouling membrane surface. (**b**) Photograph of a prepared PCBU-CA membrane. (**c**) FTIR spectra of CA membrane, PCBU, and PCBU-CA membrane. (**d**) Surface protein adsorption on the CA and PCBU-CA membrane surface. ** *p* < 0.01. AFM image and pore size distribution of the (**e**) CA and (**f**) PCBU-CA membranes.

A schematic illustration of the PCBU-CA membrane preparation procedure is shown in Figure 1a. Details regarding the PCBU synthesis method and the modified membrane fabrication process have been reported in our previous publications [20]. Briefly, polycarboxylate polyurethane (PCBU) was synthesized via a one-pot reaction with a 1:1 ratio of diethanolamine ethyl acrylate:1.6-diisocyanathohexane, followed by CH_3_OH quenching and precipitation. The chemical structure of the synthesized PCBU was confirmed by 1H NMR and ^13^C NMR (DPX400, Bruker, Billerica, MA, USA) (Figure S1). The molecular weight and polydispersity of the PCBU were evaluated using gel permeation chromatography (Waters 2414, Waters, Milford, MA, USA), as shown in Figure S2. A CA membrane filter with a cut-off size of 0.8 µm (average pore size of 0.4 µm) was plasma-initialized in a plasma cleaner (PDC-001-HP, Harrick Plasma, Ithaca, NY, USA) under 45 W of radio-frequency power for 2 min. After 5 wt% PCBU in methanol solution circulated through the CA membrane on each side, the prepared PCBU-CA membrane was dried in a vacuum oven at 40 °C overnight. The PCBU-CA membrane was then immersed in PBS buffer at a pH of 8.5 for 2 h for hydrolysis of the beta-amino ester of PCBU to generate zwitterionic carboxy betaine functional groups, which provide superior antifouling properties. The surface fibrinogen adsorption was measured by a fluorescent method [20,26]. For the investigation of membrane surface morphology, we utilized atomic force microscopy (AFM) sourced from AFM Workshop, Signal Hill, CA, USA. The AFM analysis was carried out under ambient conditions in tapping mode with a typical scan length of 15 μm and a scan rate of 0.1 Hz. Additionally, the root-mean-square roughness (Rq) was examined to compare the surface roughness of the PBCU-CA membrane with that of the CA membrane. The chemical composition of the PCBU-CA membrane was confirmed by Fourier-transform infrared spectroscopy (FT-IR; Vertex 80, Bruker, Billerica, MA, USA) and X-ray photoelectron spectroscopy (XPS; AXIS-165, Kratos, Manchester, UK). The amount of PCBU (8.3 wt%) on the CA membranes was confirmed by TGA analysis (Figure S4).

### 2.3. Virus/Plasma Separation Design and Assembly

A semi-transparent acrylic device with dimensions of 2.4 (W) × 2.4 (L) × 13.5 (H) cm was fabricated by a three-dimensional (3D) printer (Prusa SL1S, Prusa Research). The device consisted of three primary components: a blood inlet, a membrane separation chamber, and a plasma outlet. The blood inlet component has an inlet tunnel on the top for blood sample loading and a chamber with a capacity of 10 mL for the injected blood. The plasma outlet has a 5 mL capacity chamber for the filtrated plasma. The middle component is a membrane separation chamber that can be loaded with four PCBU-CA membranes for plasma filtration. Each stack in the middle chamber has a window with dimensions of 1.6 (W) × 5.0 (H) cm, and the total effective filtration area of the middle chamber is 32 cm^2^. Mesh support was included in the middle chamber to mechanically support the PCBU-CA membrane. During the filtration test, a commercial vacutainer blood collection tube (BD Vacutainer, BD Biosciences, Franklin Lakes, NJ, USA) was connected to the bottom chamber to collect plasma. Only a vacuum inside the blood collection tube was used as the driving force for plasma separation, without any external pressure.

### 2.4. Phage Separation Test

T7 phage was used as a model virus. A T7 phage suspension was dispersed in whole porcine blood to prepare phage-spiked blood with a concentration of 1 × 10^5^ plaque-forming units per mL (pfu/mL). After 10 mL of whole-phage-spiked porcine blood was manually loaded into the blood inlet chamber with a syringe, the device was connected to a Vacutainer^®^. The negative pressure in the plasma outlet chamber drove the plasma with the viral particles through the membrane to the bottom outlet chamber, while the membrane retained blood cells in the separation chamber. The separated plasma was collected in the BD Vacutainer^®^ and used for further analysis. The plasma filtration rate was periodically recorded, and the phage recovery ratio was calculated by the following equation:

$$R_{phase}=\left(\frac{C_{p}}{C_{f}}\right)\times 100\%$$

where *C_p_* and *C_f_* (pfu/mL) are the viral particle concentrations of the permeate and feed sides, respectively.

The hemoglobin level in the separated plasma was evaluated using a UV-2600 spectrophotometer (Shimadzu, Kyoto, Japan). An Olympus IX81 microscope (Olympus, Tokyo, Japan) was used to verify the absence of blood cells in the extracted plasma.

### 2.5. Phage Recovery Quantification

A quantitative plaque assay and RT-PCR were employed to test the separation of viral particles across the membrane from whole blood. The plaque assay can effectively show the recovery of infectious phages, and the result was quantitatively reinforced by RT-PCR measurements.

In this study, *E. coli* BL21 cells were used as the host of T7 phage. *E. coli* cells in 10 mL of lysogeny broth (LB) medium were cultured at 37 °C with a shaking speed of 250 rpm. When the *E. coli* concentration reached the optical density at 600 nm (OD_600_) = 0.4, the medium was diluted 50 times with fresh LB medium to prepare the *E. coli* solution for a plaque assay. A 0.1 mL volume of each sample containing T7 phage (spiked blood, centrifuged plasma, and plasma permeate from the membrane separation device) was mixed with 0.1 mL of *E. coli* solution and incubated at room temperature for 15 min. Afterward, 3 mL of soft LB agar (5 g of agar per liter) was added. The system was gently mixed and immediately poured onto prewarmed LB plates. The overlay was spread across the plate by tilting and rotating the plate until the overlay was evenly distributed. After incubating the plates for 18 h at 37 °C, we captured pictures of the culture plates and measured the viral counts. We then compared the viral counts of the plasma extracted from the membrane separation device with those of the spiked blood and centrifuged plasma.

The plasma solution samples extracted from the centrifuge and the membrane separation device were also analyzed by RT-PCR based on a previously reported protocol [27]. The plasma sample was first diluted 20-fold with DNase/RNase-free water. Next, 200 µL of the diluted solutions was mixed with 5 units (2 µL) of DNase I and incubated at 37 °C for 10 min in a heated dry bath. The tubes were then incubated at 100 °C for 15 min in a heated dry bath to denature the phage. The prepared sample was cooled to room temperature. Afterward, the sample was mixed with PowerUp™ SYBR™ Green Master Mix, 5 µM primer, and DNase/RNase-free water. Five replicates were prepared for each sample. The samples were then tested by RT-PCR with the following conditions: one cycle at 50 °C for 2 min, one cycle at 95 °C for 2 min, and 40 cycles of 95 °C for 15 s and 60 °C for 1 min.

## 3. Results and Discussion

### 3.1. Membrane Preparation and Characterization

Membrane biofouling is a critical issue in membrane biomolecule separation (e.g., ultrafiltration), as it greatly compromises the filtration efficiency of the separation process. In particular, in the separation of plasma from protein-rich whole blood, the membrane surface and pores can quickly be covered by blood proteins, followed by the adhesion of a series of other biomolecules [28]. Such biofouling can cause clogging of the membrane pores and significantly reduce the membrane flux. To rapidly collect a high volume of plasma permeate in our device, our separation device utilizes an ultralow-fouling PCBU-CA membrane (Figure 1a) [20]. The prepared PCBU-CA membrane appears as a smooth, flat white sheet (Figure 1b). Of note, the PCBU coating on the CA membrane is achieved via hydrogen bonding and electrostatic interactions [29,30]. The surface PCBU layer on the CA membrane was analyzed using FT-IR (Figure 1c). Compared to the uncoated CA membrane, the PCBU-CA membrane shows an N-H stretching band at 3324 cm^−1^ and an N−H bending band at 1540 cm^−1^. These characteristic bands confirm the presence of the PCBU layer on the PCBU-CA membrane surface. A uniform PCBU layer with a thickness exceeding 10 nm on the CA membrane surface was confirmed by XPS [31]. The presence of zwitterions on the PCBU-CA membrane surface was affirmed by the detection of the quaternary ammonium group [32]. The related data can be found in Figure S3.

We employed a fibrinogen protein adsorption test on the membrane surface to investigate the antifouling properties of the PCBU-CA membrane. Fibrinogen is a major protein in blood with high adhesion and the potential to form high-strength crosslinked fibrin on the membrane surface. Thus, fibrinogen was chosen as an in vitro screening tool for evaluating the antifouling properties of the PCBU-CA membrane. Here, we used the CA membrane as a reference for comparison. The protein adsorption of the pristine CA membrane was found to be as high as 100%, consistent with previous studies [20]. The protein adsorption of the PCBU-CA membrane was reported relative to the CA membrane. As shown in Figure 1d, the PCBU-CA membrane exhibits 58.5% less surface protein adsorption than the pristine CA membrane, indicating that the PCBU modification can effectively reduce surface fouling and improve membrane performance. The antifouling performance of our PCBU-CA membrane benefits from the zwitterionic coating layer on the surface, which can form a dense hydration layer on the membrane surface via ionic solvation [26,33]. The hydration layer can protect the membrane surface from adsorption and fibrinogen fouling [34,35,36].

We also investigated the surface morphology and pore structure of the membranes by AFM. Figure 1e indicates that the pristine CA membrane surface presents a 3D interconnected porous structure with an average pore size of 0.42 µm, which is typical for CA membranes prepared by phase inversion [37]. As depicted in Figure 1f, the PCBU-CA membrane also shows a 3D interconnected pore morphology and a pore size distribution similar to that of the pristine CA membrane, without any substantial differences. In addition, the mean pore size of the PCBU-CA membrane is 0.42 ± 0.02 µm, similar to that of the CA membrane (0.42 ± 0.01 µm). In addition, the surface roughness in the non-porous area of the PCBU-CA membrane (Rq = 29.01 nm) is comparable to that of the CA membrane (Rq = 28.64 nm). These AFM results suggest that the PCBU antifouling layer has been uniformly coated on the CA membrane without any agglomerated or localized coating domains. This uniform coating is highly advantageous for our blood–plasma separation device.

### 3.2. Plasma Separation Performance of the PCBU-CA Membrane Device

**Figure 2:**
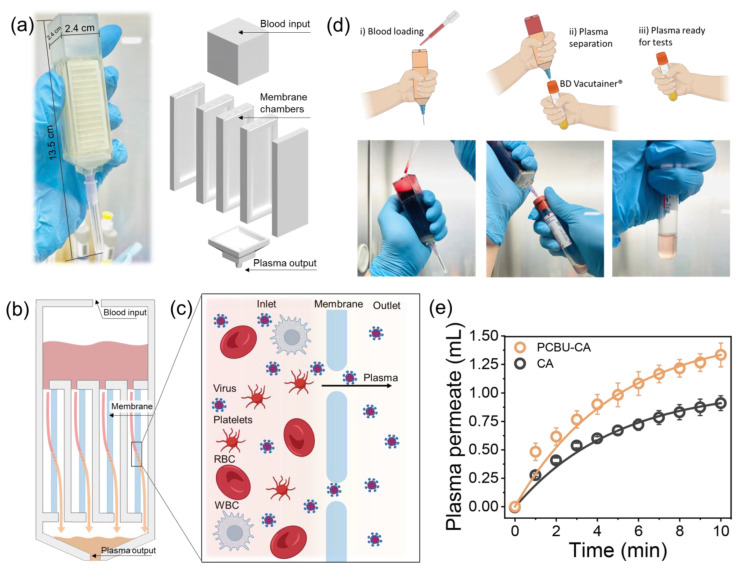
(**a**) Photograph and layout of the device for virus separation from whole blood. The device consists of a blood inlet chamber, chambers for membrane loading, and a plasma outlet chamber. (**b**) Schematic of the cross-section of the assembled device and the blood filtration path. (**c**) Illustration of the plasma separation process. The membranes reject blood cells while viruses are allowed to pass. (**d**) Illustration of the virus separation process. (**e**) Volume of plasma permeation from undiluted blood for the plasma separation device with the PCBU-CA membrane or pristine CA membrane under a vacuum pressure of 5 psi. Error bars correspond to the separated plasma volume obtained in three different experiments.

Figure 2a shows a photograph and schematic of the plasma separation device, which was designed to be portable with an easy-to-use process, not requiring specific training or additional specialized equipment. The device has dimensions of 2.4 (L) × 2.4 (W) × 13.5 (H) cm, and ideally, one should be able to easily operate the device with one hand. Compared with other membrane-based plasma separation devices consisting of single flat-sheet membranes [22,38,39,40], we employed a simple plate-and-frame membrane module configuration to enhance the membrane’s effective filtration area per volume without significantly increasing the device size. This large membrane surface area packing density (957.9 m^2^/m^3^ in the middle chamber) can rapidly yield a large volume of plasma without the need for additional power or vacuum as a driving force. In addition, to further reduce surface fouling and maintain a high plasma flux through the PCBU-CA membrane, the membranes are loaded vertically in the plasma separation device, as shown in a schematic illustration of the device cross-section (Figure 2b,c). After the whole blood was loaded in the top chamber, the whole blood flowed into four chambers in the middle component, with each chamber containing one PCBU-CA membrane. Then, the blood was sieved through the PCBU-CA membranes, which effectively retained large blood cells (red blood cells [RBCs], white blood cells, and platelets) but allowed plasma and the phase to pass. The plasma permeate was collected in a BD Vacutainer connected to the bottom chamber of the device.

Figure 2d illustrates the overall plasma separation process of our PCBU-CA membrane device. First, the pressure of the permeate side (plasma chamber and BD Vacutainer) was maintained at −5 psi. Our previous study showed that −5 psi of transmembrane pressure is an optimal driving force that causes no significant hemolysis or concentration polarization [20]. Then, 10 mL of phage-spiked blood was loaded into the blood chamber with an injection syringe or pipette (Step i). After injection, whole blood started to flow into the four middle chambers. Once blood contacted the PCBU-CA membrane, plasma started to permeate through the membrane due to the driving force of negative pressure on the permeate side (−5 psi), and the plasma started to fill the four plasma chambers in the middle part. Then, the collected plasma in the middle chambers flowed to the plasma output component, driven by gravity. The device was attached to a vacutainer, and its vacuum pressure drove the plasma directly to the vacutainer (Step ii). The device operated until the permeated plasma reached the desired volume. Then, the plasma collected in the vacutainer was ready for subsequent analysis (Step iii). Figure 2e shows the separated plasma volume collected by the plasma separation device as a function of time. The separated plasma solution was observed immediately after blood was loaded in the inlet chamber, with a rapid yield observed in the first minute. After 5 min, the blood plasma transport rate gradually decreased, possibly due to a decrease in driving force (i.e., a decrease in the vacuum level in the bottom chamber and vacutainer). The plasma volume collected by the device loaded with the PCBU-CA membrane reached 1.33 ± 0.10 mL in 10 min. Compared with the device with the pristine CA membrane (0.92 ± 0.13 mL), the PCBU-CA membrane enhanced the plasma separation performance by 46%. This result indicates that the zwitterionic PCBU coating can greatly improve the antifouling properties of the CA membrane owing to its lower protein adsorption and surface cell attachment properties [20]. Current traditional virus load assays generally require 250 µL to 1 mL of plasma, while state-of-the-art POC HIV assays with the desired sensitivity (<200 copies/mL) require at least 1 mL of plasma for a single test [1,41]. Thus, the 1.33 mL yield of undiluted plasma obtained in 10 min from the PCBU-CA membrane-loaded plasma separation device can satisfy most clinical requirements.

**Figure 3:**
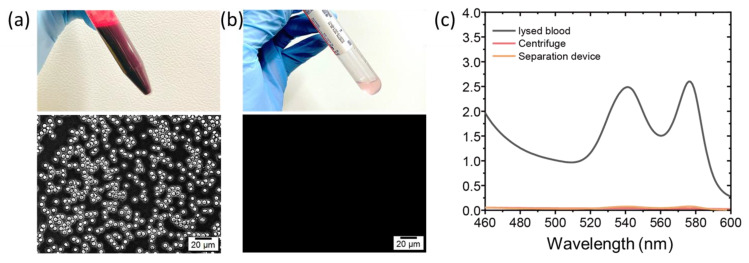
Photograph (**top**) and optical microscopy image (**bottom**) of (**a**) whole blood before loading and (**b**) collected plasma permeate. The plasma permeate reveals an absence of blood cells. (**c**) Spectrophotometer scanning of lysed blood, centrifuged plasma, and plasma separated at different vacuum pressures. The hemolysis level is represented by the absorbance at 540 and 574 nm.

Figure 3a,b show photographs and optical microscopy images of the whole blood input and the plasma solution extracted by our device. A 10 mL input of whole blood with an opaque, dark red color yielded a total of 3 mL plasma solution with a very light pink color after the separation process. Optical microscopy results of the whole blood clearly show blood cells with a size range of 3–8 µm. In contrast, the microscopy image of the plasma permeate indicates the absence of blood cells, clearly demonstrating that our plasma separation device with the PCBU-CA membrane can effectively reject blood cell components while allowing only plasma protein to pass through. In addition to fouling issues in membrane-based plasma separation, another undesired effect is hemolysis. Due to the high shear force and poor biocompatibility of most commercial membrane materials, RBC lysis can occur, releasing unwanted intracellular components. To evaluate the hemolysis level of plasma solution obtained from our plasma separation device with the PCBU-CA membrane, we measured the hemoglobin concentration using ultraviolet–visible spectroscopy and compared it with that of centrifuged plasma and lysed blood as a reference. Hemoglobin typically shows absorbance peaks at 540 and 574 nm. As shown in Figure 3c, the lysed blood shows two clear peaks, indicating the presence of hemoglobin. However, there is no notable difference between the plasma solutions obtained by centrifugation and our plasma separation device. The weak absorbance peaks at 540 and 574 nm mainly correspond to the natural hemolysis of RBCs during blood storage [21]. The hemoglobin release results demonstrate that our plasma separation device can provide high-quality separated plasma with a low hemolysis level.

### 3.3. Phage Recovery

**Figure 4:**
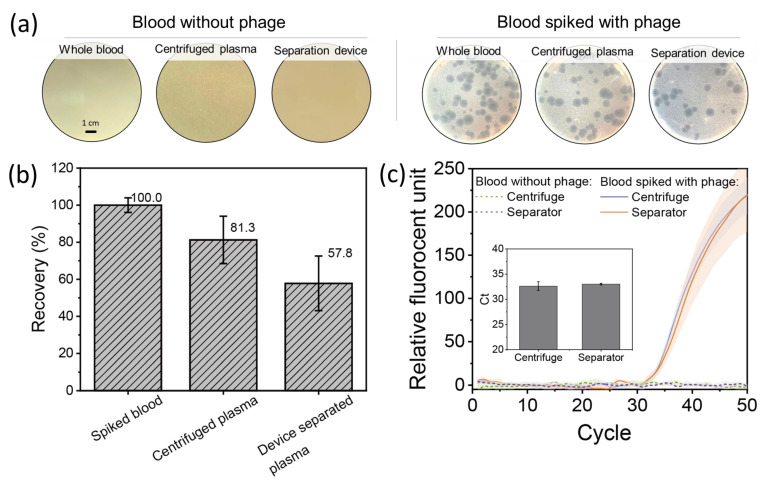
(**a**) Plaque assay images for spiked blood, centrifuged plasma, and plasma extracted by our separator with a virus loading concentration of 1 × 10^5^ pfu/mL and no virus loading. (**b**) Virus recovery of centrifuged plasma and plasma filtrated by our device. Here, spiked blood with a virus loading concentration of 1 × 10^5^ pfu/mL was used as the feed and set as a reference (100%). (**c**) Comparison of the recovery efficiency of phage virus via centrifugation and our virus separation device as a function of the amplification cycle.

As the purpose of this study was to provide plasma serum containing enough infectious viruses for accurate POC viral assay analysis, we investigated the recovery of phage during the plasma separation process. We used the T7 phage as a probe to assess the virus recovery performance of our plasma separation device, as the size of the T7 phage is similar to that of disease-causing viruses (e.g., HIV, COVID, and Zika), and it can be easily quantified by plaque assays and RT-PCR [42]. In this work, the T7 phage was dispersed in whole blood to prepare blood samples with a viral concentration of 1 × 10^5^ pfu/mL, and plasma samples obtained by the PCBU-CA membrane separation device were compared with spiked blood and plasma separated by a typical centrifuge method. Figure 4 shows the results of the phage recovery test. Of note, the phage loading in spiked blood was used as a reference, and the phage recovery (ratio of viral particle loading in permeate plasma vs. feed-spiked blood) was calculated as described in the Section 2. Plasma from whole blood without phage spiking was used as a negative control. As shown in Figure 4a, the pristine whole blood sample, centrifuged plasma, and plasma extracted via our device from pristine blood show no plaque formation. In comparison, phage plaques formed in spiked blood and the plasma samples obtained by the centrifuge method and our plasma separation device. The plasma filtered by our device with the PCBU-CA membrane showed a phage recovery of 57.8%, while the centrifuged plasma exhibited a phage recovery of 81.3%, as shown in Figure 4b. Nonetheless, the phage recovery performance of our device is in a range similar to that of other membrane-based plasma separation devices reported in the literature [19,38,39]. Compared with the PCBU-CA membrane, the device with the unmodified CA membrane showed permeated plasma with a similar viral loading (Figure S5). However, the total extracted viral amount from the device with the unmodified CA membrane was 32% less than that of the device with the PCBU-CA membrane due to lower plasma permeability. As the sizes of common disease-causing viruses (e.g., HIV, COVID, and Zika) are similar to the T7 phage, we expect comparable viral recovery rates for blood containing these other viruses. The slightly lower phage recovery of our separation device may be due to nonspecific binding on the inner surfaces of the separation device. Although the PCBU-CA membrane provides lower nonspecific adsorption [20], there is still a loss of phage that comes from the adsorption on the device surface [43]. In future work, we plan to functionalize the surface of the device with PCBU to reduce virus binding and increase virus recovery efficiency [19].

Compared with plaque assays, RT-PCR has become a promising method for detecting and quantifying viral particles due to its considerable sensitivity. We tested the suitability of plasma samples extracted with our plasma separation device for RT-PCR, using pristine whole blood and centrifuged plasma as a reference. Figure 4c depicts the fluorescence intensity as a function of the amplification cycle. The negative control (blood without phage and plasma extracted) produced almost no signal throughout the amplification process, indicating negligible amplicon production. Meanwhile, when phage-spiked blood was used as the feed solution, the fluorescence signal of the plasma serum extracted with our PBCU-CA membrane separation device showed an intensity comparable to that of centrifuged plasma. The cycle threshold (C_t_) is the amplification cycle at which the fluorescent signal increases above a predetermined baseline level, which is used to quantify the viral loading in the sample. The C_t_ of plasma extracted with our separation device is <1.3% lower than that of the centrifuged plasma. The resolution in traditional quantitative PCR is typically 1 C_t_, which can lead to a variation of 0.5–2 fold in quantitative results. Because the difference in phage recovery between filtrated and centrifuged plasma is smaller than the error range of RT-PCR, the resulting RT-PCR curves of the two plasma samples had no statistically significant difference. Combined with the plaque assay results, the RT-PCR results demonstrate that our separation device is suitable for nucleic acid amplification assays and has great potential for POC viral diagnosis.

## 4. Conclusions

In this study, we demonstrated a portable, easy-to-use, disposable membrane-based separator for real-time isolation of large volumes of plasma from whole blood. The plasma separation device loaded with a PCBU-CA membrane can yield 1.33 mL of plasma from whole blood within 10 min and a total of 3 mL of plasma after 150 min. The extracted plasma was proven to be cell-free with a low hemoglobin level. Compared with a pristine CA membrane (0.92 ± 0.13 mL), the PCBU-CA membrane shows a 46% increase in plasma separation performance, clearly indicating that the zwitterionic PCBU coating can greatly improve the antifouling properties of the CA membrane owing to its lower protein adsorption and surface cell attachment properties. A virus recovery test with whole blood and the T7 phage demonstrated that our plasma separation device is suitable for sample preparation for traditional or POC viral assays. Plaque assays showed a viral recovery of 57.8% compared with spiked blood. In addition, RT-PCR results indicate that the plasma extracted with our separation device has a virus recovery similar to that of centrifuged plasma. The plasma separation device loaded with the antifouling PCBU-CA membrane described herein can be used as a disposable, stand-alone plasma separation device with minimal lab-scale medical diagnostic instruments, enabling rapid, simple, and accurate viral diagnostics at the clinic/bedside in resource-restricted settings.

## Data Availability

Data available upon request.

## Supplementary Materials

The following supporting information can be downloaded at: https://www.mdpi.com/article/10.3390/membranes13050524/s1, Figure S1. (a) ^1^H and (b) ^13^C NMR spectrum for PCBU; Figure S2: GPC data of PCBU.; Figure S3: XPS spectra of (a) CA membrane wide scan, (b) PCBU-CA membrane wide scan, and (c) N1s high-resolution scan of non-zwitterionic and zwitterionic PCBU-CA membrane.; Figure S4. (a) TGA and (b) DTG curves of CA membrane, PCBU, and PCBU-CA membrane.; Figure S5. (a) Virus recovery of spiked blood with a virus loading concentration of 1 × 10^5^ pfu/mL, centrifuged plasma, plasma filtrated by our device with CA membrane, and plasma filtrated by our device with PCBU-CA membrane. (b) Comparison of the recovery efficiency of phage virus via centrifugation and our virus separation device with CA or PCBU-CA membrane as a function of the amplification cycle.
