# Supplementary material for: Simple Bioparticle Filtration Device Based on an Ultralow-Fouling Zwitterionic Polyurethane Membrane for Rapid Large-Volume Separation of Plasma and Viruses from Whole Blood
Source: Membranes (Basel). 2023 May 17;13(5):524. doi: 10.3390/membranes13050524 (PMC10223665; doi:10.3390/membranes13050524)
Supplement: Supplementary file 1 [file membranes-13-00524-s001.zip › membranes-2350627-supplementary.pdf]

Supplementary Information

Simple Bioparticle Filtration Device Based on an Ultralow-Fouling Zwitterionic Polyurethane Membrane for Rapid Large-Volume Separation of Plasma and Viruses from Whole Blood

Kun Wang [1], Hyang Seol [1], Alex Cheng [1,2], Nash McKeague [1,3], Megan Carlson [1], Wade Degraff [1], Sijia Huang [4] and Sangil Kim [1,*]

[1] Department of Chemical Engineering, University of Illinois at Chicago, Chicago, IL 60607, USA
[2] New Trier High School, New Trier, IL 60093, USA
[3] University of Chicago Laboratory Schools, Chicago, IL 60637, USA
[4] Lawrence Livermore National Laboratory, Livermore, CA 94550, USA; huang55@llnl.gov
* Correspondence: sikim@uic.edu

1. NMR

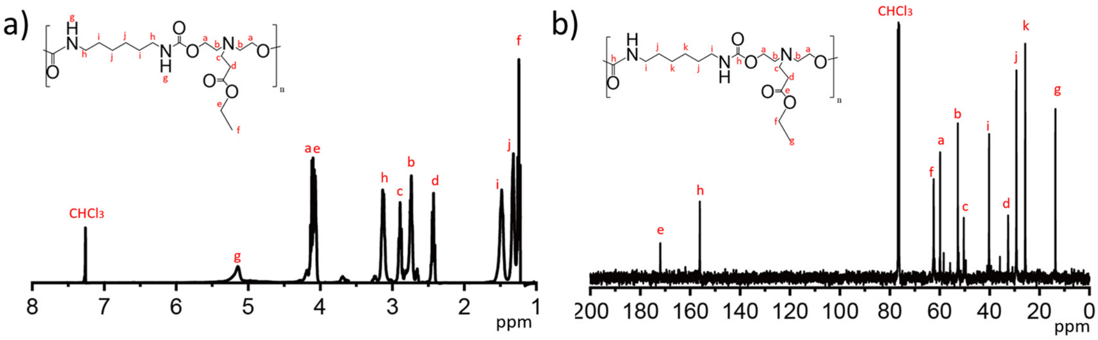

Figure S1. a) ^{1}H and b) ^{13}C NMR spectrum for PCBU.

2. GPC

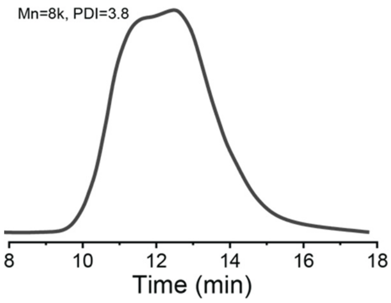

Figure S2. GPC data of PCBU.

3. X-ray photoelectron spectroscopy (XPS)

XPS (Kratos AXIS-165) was employed to study the surface of PCBU-CA membrane. The pristine CA membrane shows a primary composition of C and O, with trace amounts of N and S originating from nitrocellulose and other impurities as shown in **Figure S3a**. In the case of the PCBU-CA membrane (**Figure S3b**), the elemental N/C ratio of 3/16.6 is close with the theoretical N/C ratio in bulk PCBU (3/17), suggesting a uniform PCBU layer on the CA membrane surface. The thickness of the PCBU layer is estimated to be close to or greater than the penetration depth (~10 nm) of XPS beam, which is consistent with our previous calculation (5 to 20 nm) derived from TGA data [1].

In the final stage of PCBU-CA membrane preparation, the beta-amino groups in the PCBU layer underwent hydrolysis to generate zwitterions. To evaluate the presence of zwitterions on the surfaces of PCBU-CA, high-resolution N1s scans were compared between unhydrolyzed and hydrolyzed PCBU-CA. As shown in **Figure S3c**, the quaternary ammonium group was observed after hydrolysis, indicating successful hydrolyzation and the generation of zwitterions on the PCBU-CA membrane surface.

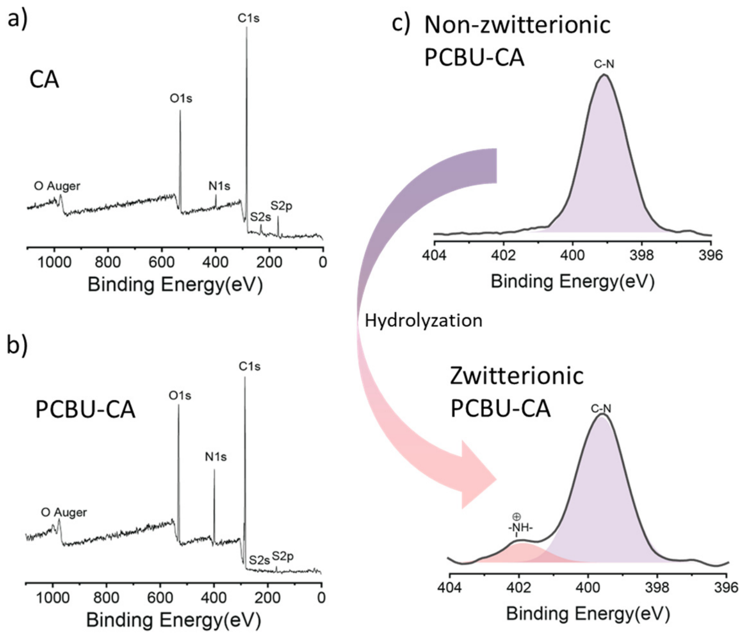

Figure S3. XPS spectra of a) CA membrane wide scan, b) PCBU-CA membrane wide scan, and c) N1s high-resolution scan of non-zwitterionic and zwitterionic PCBU-CA membrane.

4. Thermogravimetric analysis (TGA)

TGA (PerkinElmer, USA) was performed on the CA membrane, PCBU film, and PCBU-CA membrane. The temperature gradually increased from 50 to 700 °C at a rate of 5 °C/min in an air atmosphere, and the temperature was held at 110 °C for 180 min to remove any moisture. **Figures S4a and S4b** show a quantitative analysis of the PCBU coating layer on the membrane. Compared with CA membrane, the PCBU-CA membrane shows a notable increase in the weight loss peak at 240 °C, which corresponds to the PCBU layer on the membrane. The comparison of these TGA curves verifies the existence of a PCBU layer on the membrane. These TGA curves confirm the existence of a PCBU layer on the membrane. By comparing the weight loss peak at 240 °C between the PCBU-CA membrane and PCBU, we quantified the amount of PCBU on the surface of the PCBU-CA membrane as 8.3 wt %.

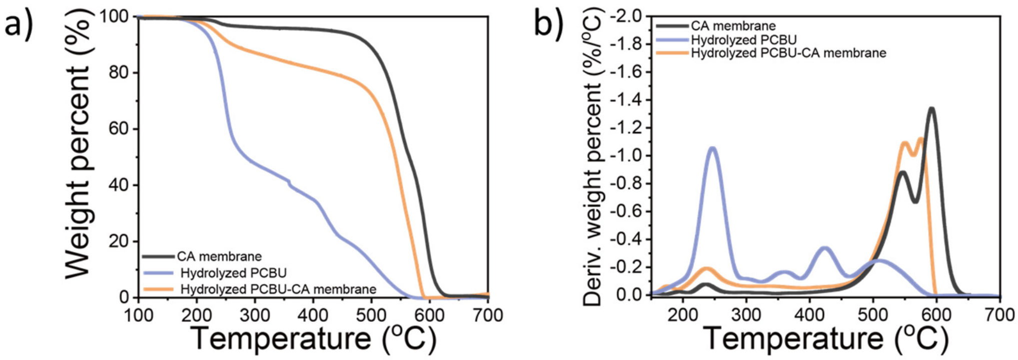

Figure S4. a) TGA and b) DTG curves of CA membrane, PCBU, and PCBU-CA membrane.

5. Phage recovery of CA membrane

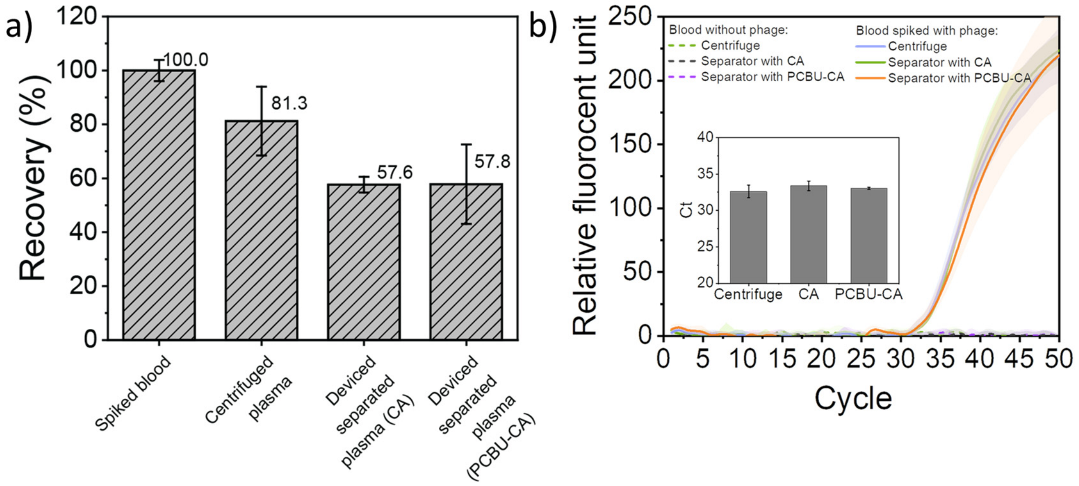

Figure S5. a) Virus recovery of spiked blood with a virus loading concentration of 1 × 105 pfu/mL, centrifuged plasma, plasma filtrated by our device with CA membrane, and plasma filtrated by our device with PCBU-CA membrane. b) Comparison of the recovery efficiency of phage virus via centrifugation and our virus separation device with CA or PCBU-CA membrane as a function of the amplification cycle.

1. Wang, K.; Seol, H.; Liu, X.; Wang, H.; Cheng, G.; Kim, S. Ultralow-Fouling Zwitterionic Polyurethane-Modified Membranes for Rapid Separation of Plasma from Whole Blood. *Langmuir* **2021**, *37*, 10115-10125, doi:10.1021/acs.langmuir.1c01477.
